# Prevalence and Predictors of Caregiver Distress in Six European Communities: Data From the IBenC Study, Using interRAI‐Home Care Assessments

**DOI:** 10.1111/scs.70005

**Published:** 2025-03-14

**Authors:** Inga Valgerdur Kristinsdottir, Palmi V. Jonsson, Ingibjorg Hjaltadottir, Kristin Bjornsdottir

**Affiliations:** ^1^ Faculty of Nursing and Midwifery University of Iceland Reykjavik Iceland; ^2^ The Primary Health Care of the Capital Area Reykjavik Iceland; ^3^ Faculty of Medicine University of Iceland Reykjavík Iceland; ^4^ Geriatric and Rehabilitation Services Landspítali/National University Hospital Reykjavik Iceland

**Keywords:** ageing, caregiver distress, family caregiver, home care, informal caregiver, interRAI‐home care, older people

## Abstract

**Background:**

In a changing world where populations are ageing and older people need assistance to live at home, caring for an older relative can be challenging and have various consequences for caregivers.

**Methods:**

In this cross‐sectional study, caregiver distress in six European countries—Iceland, Belgium, Finland, Germany, Italy and the Netherlands—was examined and compared. The study aimed to determine the prevalence of distress among caregivers of older people receiving home care in these six countries and identify if factors related to the older person's condition, such as health or function, predict it. The analysis drew on data collected from 2014 to 2016 for the IBenC study (Identifying Best Practices for care‐dependent elderly by Benchmarking Costs and Outcomes of Community Care), using the interRAI‐Home Care (HC) instrument. A total of 2884 home care clients > 65 years from the six countries participated in the study. Descriptive statistics indicated the characteristics of the sample, and bivariate and multivariate logistic regression models established predictive independent variables for caregiver distress.

**Results:**

The percentage of caregiver distress was highest among Icelandic caregivers (34%). In the other countries, it varied from 9% to 22% and was lowest in Finland. Caregivers of clients with signs of depression, clients who have bladder incontinence or who had stayed in hospital in the last 90 days were more likely to experience caregiver distress. Caregiver distress was more prevalent if a client was at risk of severe health decline and had increased care needs.

**Conclusion:**

Using data from interRAI‐HC assessments makes it possible to relate indications of caregiver distress to the characteristics of the older person cared for. Hence, improving their condition might have favourable effects on caregivers. Alertness to caregiver distress is crucial.

## 1 Introduction

In recent decades, policymakers have emphasised ‘ageing in place’ or remaining living in the community despite needing support and care rather than moving to nursing homes. Reflecting the significance of ageing in place is a statement from the OECD (The Organization for Economic Cooperation and Development) suggesting that those needing care should be enabled to continue living in their homes [1]. Governments worldwide have encouraged older people to lead independent lives, resulting in an increased number of older people with care needs who remain living in their homes [2, 3] and receiving assistance from formal and informal caregivers [4].

Care for infants and frail older people is essential in all societies; thus, considering what it entails and how it can best be arranged is necessary. Caring for an older family member has become more onerous in recent decades. The caregiver role has become more complex and longer lasting because of medical advances, shorter hospital stays and increased longevity [5]. Informal caregivers usually take on the caregiving role unsolicited and consider themselves ready for the task. Despite their willingness and positivity, they may be unprepared for the physical, emotional, psychological and financial burdens of the caregiving role [6], and they may not possess the appropriate skills to provide care [7]. Caring for an older person with multimorbidity, impaired functional ability and complex care needs can be especially overwhelming and lead to caregiver distress [8, 9, 10].

Following the demographic changes described above, many older people are living with more complicated care needs, and the caregiver role has become more complex which has called for rethinking of services provided to frail older people living at home. The United Nations (UN) and the World Health Organization (WHO) developed a strategic plan for healthy ageing, which means creating an environment and opportunities that enable people to be and do what they value throughout their lives and have functional abilities and capabilities that enable them to be and do what they have reason to value. The years 2021–2030 were designated as The UN Decade of Healthy Aging [11].

This plan has influenced the development of services for older people around the world. Governments in various countries have identified service needs and the services provided. As the World Health Organization [11] notes, considering the situation in various countries to realise what must be done to improve care services designed for older people is essential.

Although the idea of ageing in place is usually considered positive, it has meant that much work has been transferred from formal service to older people and their families. Family caregivers, often referred to as informal caregivers since they are unpaid, provide extensive support to older people, making it possible for them to stay at home. In many situations, they may even provide advanced health care and become the older people's case managers and advocates, filling the gaps that often appear in care systems characterised by fragmented services. Informal caregivers are generally family or friends, especially spouses or adult children, as well as children‐in‐law or neighbours of the care recipients [12]. Most informal caregivers are female; they are wives, daughters and daughters‐in‐law [13]. It has been estimated that informal caregivers provide 60%–90% of home care [10].

Numerous studies have shown that being an informal caregiver can be stressful and lead to the feeling of burden. The term ‘caregiver distress’ was coined to describe this situation. Distress has been defined as discomfort, strain or apprehension [14], but in this article, it mainly refers to exhaustion. In studies from New Zealand [8] and Canada [10], predictors of caregiver distress were identified based on results from the older person's interRAI‐Home Care (HC) assessments, described below. The results for the two countries were comparable but were not identical. Variables increasing the odds of the onset of caregiver distress included clients´ aggressive behaviour and higher scores on the depression rating scale (DRS), cognitive performance scale (CPS) and the activities of daily living hierarchy scale (ADLH). If the caregiver was a spouse or lived with the client, the likelihood of caregiver distress similarly increased. Being a long‐term caregiver and the care recipient being physically inactive or having Alzheimer's or other related dementias also increased distress.

Identifying caregiver distress predictors in the European context, using similar methodologies to those of the previously mentioned studies, contributes to knowledge development in this increasingly important area. Individuals in demanding care roles could become clients with impaired mental, physical and social skills. Therefore, it is important to determine if any characteristics of home care clients enhance caregiver distress. Based on such knowledge, formal care services can be designed to support family caregivers at high risk of caregiver distress.

This study contributes to the literature on older people with multimorbidity living at home and home care services in Europe. It aimed to determine the prevalence of caregiver distress among informal caregivers in six European countries and identify which factors in the client's health, functional status and conditions predict caregiver distress.

## 2 Materials and Methods

### 2.1 Study Design and Sample

In this descriptive cross‐sectional study, data from a European study titled Identifying best practices for care‐dependent elderly by Benchmarking Costs and outcomes of Community Care (IBenC) were used. The data were collected in six European countries—Iceland, Belgium, Finland, Germany, Italy and the Netherlands—from 2014 to 2016 [15]. The interRAI‐HC assessment was used to collect data from older persons receiving home care services. Methodologies and the sample description of the IBenC study have been previously published [15, 16, 17].

The participating home care clients were 65 years or older and remained in care for at least 6 months after initiating participation. Excluded from the study were clients who were at the end stage of life, had planned admittance to a nursing home within 6 months, had received care for a short time and clients who were both diagnosed with moderate or severe cognitive impairment (CPS score ≥ 3) and without a known informal caregiver or legal representative. It was considered important that mentally incompetent persons (scoring ≥ 3 on the CPS scale) had a close relative, legal representative or legal guardian who was competent to provide informed consent on behalf of the home care client and could provide reliable information on clients’ care utilisation. Written consent was obtained from participants according to local regulations. Informed consent was not required for clients from home care organisations that utilised interRAI‐HC as part of their routine care and were performed for clinical purposes by organisations own staff [15]. The total sample in participating countries consisted of 2884 home care clients served by 38 home care organisations. Data were simultaneously collected from three target groups: home care organisations, home care clients and home care professionals. In the present study, data from home care clients were used. Data collection followed a prospective longitudinal design with interRAI‐HC assessments conducted at baseline and again at 6 and 12 months. The analysis presented in this article drew on the data from the baseline assessment.

Home care organisations in the participating countries were selected based on the diversity of their location, size, management or form of payment; thus, the selection was based on various care practices rather than being representative of a country. Therefore, the sample's representativeness remains uncertain, except for Iceland, where the sample, drawn from the entire population in the capital, accurately represents home care clients within that locale.

The baseline data were collected simultaneously for each individual, except in Italy, where baseline data were documented retrospectively 6 months later. Thus, disability levels may have been overestimated at baseline. In the Netherlands, cognitive impairment was very low, likely because one of the main reasons for refusal during the recruitment process was cognitive impairment [15].

### 2.2 The interRAI—Home Care Instrument

This study used interRAI‐HC, a comprehensive, person‐centred, structured geriatric assessment for clients in‐home care and community‐based settings [18, 19]. This assessment tool is used internationally in health care settings for routine care to support assessment, care planning for vulnerable clients and research studies. It offers a broad overview of a home care client's sociodemographic, health, functional status, resources and service use [19].

The interRAI‐HC instrument provides a range of data that are circumscribed information about issues, including hearing, vision and activities of daily living, and outcomes from scales using information from multiple items to calculate a person's risk of a specific event. It provides outcome measures to track clients´ clinical status over time [20]. The ADLH scale is an incremental scale that evaluates functional status and highlights the loss of skills at early and later stages. Fewer points are assigned for early lost skills, such as bathing, and more points are assigned for later lost skills, such as eating. The scores range from 0 (no impairment) to 6 (total dependence) [21]. The CPS scale measures cognitive impairment using items concerning memory impairment, decision‐making about daily activities, the ability to be understood and the level of consciousness. The score ranges from 0 to 6, with a score of ≥ 3 indicating the presence of moderate to very severe cognitive impairment [22]. One of the outcomes of the interRAI‐HC is the DRS scale, a screening tool for depression. The scale uses several items relating to mood, such as making negative statements, persistent anger with self or others and repetitive anxious behaviours. The score ranges from 0 to 14, with higher scores indicating an increased risk of depression [23]. The Changes in Health, End‐Stage Disease and Signs and Symptoms Scale (CHESS) identifies care recipients with higher levels of medical complexity who are at risk of severe health decline. Items included in the scale are health conditions, end‐stage disease, nutritional issues and changes in decision‐making and ADL status. Scores on the CHESS scale range from 0 (no health instability) to 5 (very high health instability) [24]. The PAIN scale reports the presence and intensity of pain and ranges from 0 to 4, with higher scores representing greater pain. The interRAI‐HC also includes decision support algorithms, such as the method for assigning priority levels (MAPLe), an algorithm that provides composite measures by combining various factors, such as ADL impairment, cognition, falls, IADL and behaviour. It indicates older peoples' care needs, predicts long‐term care placement and may indicate caregiver distress [25].

### 2.3 Study Variables

Although the interRAI‐HC assessment mostly captures information about older people receiving home care, three items focus on the informal caregiver and have been used to assess caregiver distress [8, 10, 26, 27, 28]. The first concerns whether a caregiver can continue caring activities due to declining health; the second reflects the primary caregiver's expressed feelings of distress, anger or depression; and the third allows family and close friends to report feeling overwhelmed by the older people's illnesses. In this study, caregiver distress was identified as present if one or more of these items was recorded as true by the assessor. These three items are closely related, and it is considered important for identifying the reserves of the informal caregiver support system to include all three of them [8, 27, 28, 29].

Of the sample, 388 home care clients, constituting 13.5%, reported having no informal caregivers and were thus excluded from the analysis. Those who had informal caregivers were divided into two groups: (1) those with a caregiver who indicated caregiver distress as defined earlier, and (2) those with a caregiver who did not indicate caregiver distress. These two groups were compared on clients' sociodemographic and health characteristics from the interRAI‐HC assessment. The selection of variables to test association with caregiver distress was based on previous literature and the researchers' clinical experience. The following variables related to older people being cared for were used: age, gender, marital status, primary caregiver living with the client, physical activity over the previous 3 days and health‐related characteristics, such as nutritional difficulties, dyspnoea at rest and bladder or bowel incontinence. Outcomes from the ADLH, CPS, CHESS, DRS, MAPLe and PAIN scales were used for the comparison. The association between caregiver distress and several other factors was also examined, including the older peoples need for telephone assistance, daily monitoring by a home care nurse, hospital admission within the past 90 days, and the average number of hours of formal care received per week. The number of formal care hours was calculated by summing the hours of service provided by home care and social service entities to the client in the 7 days preceding the evaluation.

### 2.4 Data Analysis

Descriptive statistics were used to describe the characteristics of the sample, as were the proportion of people with caregiver distress. Cross‐tabulation analysis was utilised to assess the prevalence of caregiver distress across various conditions and client characteristics, applying a chi‐squared test to evaluate differences between groups with a significance level of *p* < 0.05. T‐tests were also conducted to explore the effect of continuous variables like age and hours of formal care on caregiver distress, maintaining the same significance threshold. The primary outcome, distress versus not distressed caregivers, was analysed using bivariate logistic regression to generate odds ratios and 95% confidence intervals. Variables that were statistically significant in any of the countries were considered for inclusion in the multiple logistic regression model. This multiple approach allows for a comprehensive analysis that controls for confounding factors, reveals complex relationships between variables and enhances understanding of the influence on the outcome. In this model, caregiver distress was the dependent variable, and the significant variables hypothesised to influence distress were included as independent variables. SPSS version 28 was used to conduct the analyses.

## 3 Results

Table 1 indicates the characteristics of the 2453 study participants who have an informal caregiver and how they were divided among countries. The average age from the six countries was 83.1 years. The majority of participants were female (67.3%), with the lowest percentage in Italy (57.5%) and the highest in the Netherlands (71.3%) and Finland (71.2%).

**TABLE 1:** Characteristics of home care clients who have an informal caregiver.

	Belgium		Finland		Germany		Iceland		Italy		The Netherlands		All countries	
	%	*n*	%	*n*	%	*n*	%	*n*	%	*n*	%	*n*	%	*n*
Study sample—*n*		482		379		292		417		496		387		2453
Age, years—mean (SD)	82.5	(6.7)	83.5	(6.6)	84.7	(7.0)	83.7	(7.0)	81.9	(7.9)	82.8	(7.2)	83.1	(7.2)
Female	66.8	(320)	71.2	(270)	70.5	(206)	69.8	(291)	57.5	(285)	71.3	(276)	67.3	(1648)
Married	36.6	(172)	17.7	(67)	34.2	(100)	30.7	(128)	45.0	(202)	38.1	(94)	33.9^e^	(763)
Living alone	47.6	(226)	78.1	(296)	61.0	(178)	60.9	(254)	16.3	(81)	67.4	(261)	53.0^f^	(1296)
ICG^a^ lives with the client	59.4	(262)	12.1	(46)	31.5	(92)	34.3	(143)	67.7	(336)	29.7	(115)	41.2^g^	(994)
Informal caregiver														
Spouse	30.7	(148)	11.3	(43)	26.7	(78)	26.9	(112)	30.0	(149)	26.9	(104)	25.8	(634)
Child or child‐in‐law	55.4	(267)	69.9	(265)	59.2	(173)	61.2	(255)	61.9	(307)	57.4	(222)	60.7	(1489)
Other^b^	13.9	(67)	18.7	(71)	14.0	(41)	12.0	(50)	8.1	(40)	15.8	(61)	13.5	(330)
Caregiver distress	27.7	(133)	9.2	(35)	13.7	(40)	34.1	(142)	22.2	(110)	16.8	(65)	21.4	(525)
Informal care provided^c^—mean (SD)	NA		5.9	(13.5)	8.3	(14.0)	8.8	(14.8)	23.2	(17.2)	7.9	(14.2)	11.6	(16.4)
ADLH score—mean (SD)	3.2	(1.2)	0.8	(1.4)	2.2	(1.7)	0.6	(1.1)	3.9	(1.7)	0.5	(1.2)	2.0	(1.9)
CPS score—mean (SD)	1.4	(1.6)	1.4	(1.2)	1.5	(1.7)	1.1	(1.2)	2.4	(2.1)	0.7	(0.9)	1.4	(1.6)
CHESS score—mean (SD)	1.1	(1.0)	0.7	(0.9)	0.6	(0.9)	1.2	(1.0)	1.6	(1.3)	1.3	(1.0)	1.1	(1.1)
DRS score—mean (SD)	1.8	(2.5)	1.0	(1.9)	1.5	(2.7)	1.2	(1.8)	1.3	(2.0)	1.7	(2.2)	1.4	(2.2)
MAPLe score—mean (SD)	3.5	(0.8)	3.3	(1.3)	3.4	(1.1)	3.0	(1.3)	3.7	(0.8)	2.5	(1.4)	3.2	(1.2)
PAIN scale score—mean (SD)	0.8	(0.9)	1.0	(1.0)	0.7	(0.9)	1.0	(1.0)	0.7	(0.9)	1.0	(1.2)	0.9	(1.0)
Hours of formal care^d^—mean (SD)	8.8	(7.7)	5.3	(5.3)	5.8	(5.4)	3.6	(3.8)	1.0	(2.7)	5.0	(4.9)	4.8	(5.8)

*Note:* Data are presented as percentages and numbers unless otherwise indicated.

Abbreviations: ADLH, activities of daily living hierarchy; CHESS, changes in health, end‐stage, disease and signs and symptoms; CPS, cognitive performance scale; DRS, Depression Rating Scale; MAPLe, method for assigning priority levels.

^a^ Informal caregiver.

^b^ Other = sibling, other relative, friend and neighbor.

^c^ Average hours over the last 3 days.

^d^ On average last 7 days.

^e^ Ratio of 2254 responses.

^f^ Ratio of 2446 responses.

^g^ Ratio of 2412 responses.

Just over a third (33.9%) of the participants were married, while more than half (53%) lived alone, ranging from 16.3% in Italy to 78.1% in Finland. About 41.2% of caregivers lived with the clients, 12.1% in Finland and 67.7% in Italy. In 60.7% of cases, the caregiver was a child or child‐in‐law of the client; spouses accounted for 25.8% and 13.5% had other connections. Informal caregivers in Italy provided the most care, averaging about 23 h 3 days preceding the assessment. Conversely, caregivers in Finland provided an average of nearly 6 h, with the average in other countries being around 8 h. The average score on various scales from the outcomes of the interRAI‐HC assessment varies between countries. Home care clients in Germany, Belgium and Italy had the highest scores on average on the ADL and cognitive scales, indicating they had the most impaired abilities.

**FIGURE 1:**
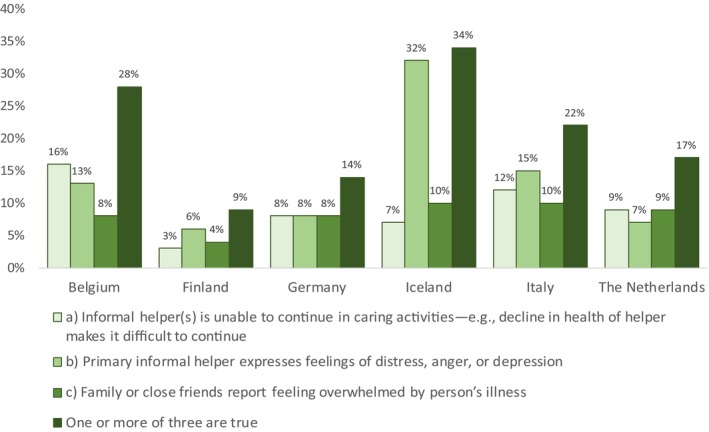
Prevalence of caregiver distress. The rate of responding YES to each statement separately and when YES was to one or more of the three statements.

Figure 1 indicates the prevalence of caregiver distress among informal caregivers caring for home care. The highest percentage was in Iceland (34%), and the second highest was in Belgium (28%), followed by Italy (22%), the Netherlands (17%) and Germany (14%) and Finland (9%). Positive responses to each of the three items concerning informal caregivers varied across countries. The highest percentage (32%) for a single item was from Iceland in response to the statement: ‘Primary informal helper expresses feelings of distress, anger, or depression’. The highest response rate (16%) to the statement: ‘Informal helper(s) is unable to continue in caring activities – e.g., decline in health of helper makes it difficult to continue’ was in Belgium, and the statement: ‘Family or close friends report feeling overwhelmed by person's illness’ had the highest response rate in Iceland and Italy (10%).

The sociodemographic and health characteristics of home care clients, stratified by clients with a distressed caregiver (WDC) or a not‐distressed caregiver (NDC) are shown in Table 2. The predictive variables for caregiver distress varied considerably between the six countries. Where caregiver distress was present, a higher percentage of home care clients were married compared to not‐distressed caregivers, reaching statistical significance in all of the countries except Finland. Additionally, a higher incidence of caregiver distress was noted when home care clients lived with the caregiver, with this finding being significant in all examined countries except Italy. Similarly, significant distress was observed in Germany, Iceland and the Netherlands when informal caregivers provided at least 10 h of care 3 days preceding the interRAI‐HC assessment. The presence of bladder or bowel incontinence in the care recipient as well as scores of three or higher on the ADLH, CPS and DRS scales or four or higher on the MAPLe scale were associated with an increased likelihood of caregiver distress. However, the statistical significance of these associations varied by country (Table 2).

**TABLE 2:** Characteristics of home care clients stratified by the presence or absence of caregiver distress.

	Belgium (482)		Finland (379)		Germany (292)		Iceland (417)		Italy (496)		the Netherlands (387)		All (2453)		Total
	WDC	NDC	WDC	NDC	WDC	NDC	WDC	NDC	WDC	NDC	WDC	NDC	WDC	NDC	
	% (*n*)	% (*n*)	% (*n*)	% (*n*)	% (*n*)	% (*n*)	% (*n*)	% (*n*)	% (*n*)	% (*n*)	% (*n*)	% (*n*)	% (*n*)	% (*n*)	% (*n*)
Age—mean (SD)	80.9** (6.4)	83.2 (6.7)	82.7 (6.8)	83.6 (6.6)	83.3 (8.1)	84.9 (6.8)	83.5 (7.2)	83.8 (7.0)	80.3* (8.0)	82.3 (7.8)	81.7 (8.0)	83.1 (7.0)	81.9** (7.4)	83.4 (7.1)	83.1 (7.2)
Female	64.1 (84)	67.7 (235)	60.0 (21)	72.4 (249)	62.5 (25)	71.8 (181)	61.3* (87)	74.2 (204)	45.5* (50)	60.9 (235)	63.1 (41)	73.0 (235)	58.9** (308)	69.5 (1339)	67.3 (1647)
Married	46.9* (61)	32.7 (111)	25.7 (9)	16.9 (58)	65.0** (26)	29.4 (74)	39.4* (56)	26.2 (72)	59.6** (59)	40.9 (143)	55.3* (26)	34.0 (68)	41.8** (237)	29.9 (526)	33.9 (763)
Primary caregiver lives with the HC‐client	69.8* (88)	55.2 (174)	25.7* (9)	10.8 (37)	57.5** (23)	27.4 (69)	41.5* (59)	30.5 (84)	73.6 (81)	66.1 (255)	44.6* (29)	26.7 (86)	55.8** (289)	37.2 (705)	41.2 (994)
Informal help ≥ 10 h last 3 days	NA		20.0 (7)	11.3 (39)	47.5** (19)	18.7 (47)	32.4** (46)	17.5 (48)	80.9 (89)	73.0 (284)	35.4** (23)	14.6 (47)	46.8** (184)	29.4 (465)	32.9 (649)
ADLH score ≥ 3	87.6 (113)	83.4 (287)	28.6* (10)	13.4 (46)	67.5* (27)	44.0 (111)	12.7* (18)	6.2 (17)	84.1 (90)	79.4 (296)	10.8 (7)	9.3 (30)	51.2** (265)	41.2 (787)	43.3 (1052)
CPS score ≥ 3	30.1** (37)	14.0 (48)	31.4** (11)	9.6 (33)	32.5 (13)	21.8 (55)	19.0** (27)	4.7 (13)	40.2 (43)	36.5 (136)	3.1 (2)	2.2 (7)	26.0** (133)	15.3 (292)	17.6 (425)
CHESS score ≥ 3	10.2 (12)	7.7 (26)	20.0** (7)	4.1 (14)	5.0 (2)	4.8 (12)	21.1** (30)	3.3 (9)	31.8 (34)	26.0 (97)	16.9 (11)	9.9 (32)	18.9** (96)	10.0 (190)	11.9 (286)
DRS score ≥ 3	37.5** (48)	21.7 (75)	37.1** (13)	11.6 (40)	42.5* (17)	19.4 (49)	28.9** (41)	11.3 (31)	32.7* (35)	19.0 (71)	43.1** (28)	21.7 (70)	35.2** (182)	17.6 (336)	21.3 (518)
Maple score ≥ 4	59.0** (62)	39.5 (120)	57.1 (20)	43.9 (150)	48.7 (19)	36.7 (91)	59.2** (84)	27.3 (75)	60.8 (31)	59.0 (82)	31.1 (19)	22.3 (69)	54.3** (235)	36.3 (587)	40.1 (822)
PAIN scale score ≥ 2^a^	14.6 (19)	20.8 (70)	25.7 (9)	28.5 (98)	20.0 (8)	23.0 (58)	34.5 (49)	32.4 (89)	21.8 (24)	20.7 (80)	33.8 (22)	33.2 (107)	25.1 (131)	26.2 (502)	26.0 (633)
Bladder incontinence	86.3* (113)	77.5 (268)	62,9* (22)	39.6 (127)	65.0* (26)	49.6 (125)	55.6** (79)	36.7 (101)	68.2 (75)	64.8 (250)	52.3 (34)	44.7 (144)	66.7** (349)	52.7 (1015)	55.7 (1364)
Bowel incontinence	62.4** (83)	45.7 (158)	17.1 (6)	14.0 (48)	45.0* (18)	22.6 (57)	14.8* (21)	7.6 (21)	47.3 (52)	46.6 (180)	18.5 (12)	12.7 (41)	36.6** (192)	26.2 (505)	28.4 (697)
Nutritional problem	34.1* (45)	21.5 (74)	0 (0)	2.9 (10)	20.0* (8)	9.5 (24)	3.5 (5)	3.6 (10)	30.9 (34)	31.1 (120)	7.7 (5)	7.8 (25)	18.5* (97)	13.7 (263)	14.7 (360)
Dyspnoea at rest	5.3 (7)	2.6 (9)	0 (0)	0.6 (2)	2.5 (1)	2.8 (7)	5.6 (8)	2.9 (8)	4.5 (5)	5.4 (21)	10.8 (7)	8.7 (28)	5.4 (28)	3.9 (75)	4.2 (103)
Hospital admission in the last 90 days	16.5 (22)	10.9 (38)	37.1* (13)	21.8 (75)	17.5 (7)	13.1 (33)	34.5** (49)	16.0 (44)	56.4* (62)	45.1 (174)	15.4 (10)	10.6 (34)	31.0** (163)	10.7 (398)	22.9 (561)
≥ 2 h of physical activities in the last 3 days	15.5 (20)	9.5 (33)	5.7 (2)	18.6 (64)	10.0* (4)	27.4 (69)	6.3* (9)	16.7 (46)	0.9 (1)	0.3 (1)	32.3 (21)	34.2 (110)	11.0** (57)	16.9 (323)	15.6 (380)
Client uses phone with assistance	47.4** (63)	27.3 (95)	34.3* (12)	16.0 (55)	32.5* (13)	19.4 (49)	12.0* (17)	4.0 (11)	70.9 (78)	64.5 (249)	7.7 (5)	6.5 (21)	35.8** (188)	24.9 (480)	27.2 (668)
Daily nurse monitoring last 7 days	76.7* (102)	80.5 (280)	5.7 (2)	11.3 (39)	47.5 (19)	61.1 (154)	2.8 (4)	2.9 (8)	2.7 (3)	0.8 (3)	12.3 (8)	5.3 (17)	26.3 (138)	26.0 (501)	26.1 (639)
Hours of formal care ‐ mean (SD)	8.9 (7.1)	8.7 (8.0)	9.2** (8.7)	4.9 (4.7)	6.7 (6.0)	5.7 (5.3)	4.4** (4.2)	3.1 (3.5)	1.5* (3.6)	0.9 (2.3)	6.5* (6.0)	4.7 (4.6)	5.7** (6.3)	4.6 (5.6)	4.8 (5.8)

*Note:* Chi‐square was used for cross‐tabs analyses, and *t*‐tests were conducted on continuous variables: Age and hours of formal care.

Abbreviations: ADLH, activities of daily living hierarchy; CPS, Cognitive Performance Scale; CHESS, changes in health, end‐stage disease and signs and symptoms; DRS, Depression Rating Scale; MAPLe, method for assigning priority levels; NDC, not distressed caregiver; WDC, with distressed caregiver.

^a^ From mild to unbearable pain.

* *p* < 0.05.

** *p* < 0.001.

Among informal caregivers with identified caregiver distress, a higher proportion of home care clients had been admitted to a hospital in the last 90 days compared to those without distress, and the difference was significant in Finland, Iceland and Italy. Home care clients received more hours of formal care, on average, when caregiver distress was present compared to when it was not, and this difference was statistically significant in Finland, Iceland, Italy and the Netherlands.

Table 3 presents the multiple logistic regression models predicting caregiver distress, which indicate the differences among the six countries. Caregivers residing with home care clients had a higher likelihood of caregiver distress, with significant associations noted in Finland, Germany and the Netherlands. Factors related to the cognitive and physical health of home care clients, including scores of three or higher on the CPS, DRS and CHESS scales and the presence of bladder incontinence, were associated with increased caregiver distress. However, the significance of these associations varied by country. Recent hospital admissions were associated with a higher incidence of caregiver distress in all countries included in the study; however, this association was statistically significant solely in Iceland. Furthermore, the duration of formal care was a significant predictor of caregiver distress in Finland, Iceland and the Netherlands, but with a low OR (1.06 in the Netherlands to 1.10 in Finland).

**TABLE 3:** Multiple regression analysis of risk factors for caregiver distress.

	Belgium	Finland	Germany	Iceland	Italy	the Netherlands	All Countries
	OR [95% CI]	OR [95% CI]	OR [95% CI]	OR [95% CI]	OR [95% CI]	OR [95% CI]	OR [95% CI]
	(*n* = 482)	(*n* = 379)	(*n* = 292)	(*n* = 417)	(*n* = 496)	(*n* = 491)	(*n* = 2453)
Age of the client	0.96* [0.92–1.00]	1.01 [0.95–1.07]	1.00 [0.94–1.05]	1.01 [0.97–1.04]	1.01 [0.96–1.06]	1.00 [0.96–1.04]	0.99 [0.97–1.01]
Client is female	0.77 [0.45–1.31]	0.54 [0.23–1.31]	0.79 [0.34–1.84]	0.46* [0.27–0.79]	0.60 [0.30–1.22]	0.73 [0.38–1.41]	0.63** [0.49–0.81]
Caregiver lives with client	1.29 [0.75–2.22]	3.20* [1.05–9.80]	3.97** [1.75–9.01]	1.18 [0.70–1.98]	1.34 [0.62–2.92]	2.56* [1.33–4.93]	1.65** [1.27–2.14]
Client DRS score ≥ 3	1.56 [0.89–2.72]	5.36** [2.13–13.46]	4.09** [1.76–9.49]	3.06** [1.67–5.61]	3.10* [1.38–6.96]	3.11** [1.65–5.86]	2.52** [1.93–3.29]
Client CPS score ≥ 3	1.48 [0.74–2.94]	3.63* [1.18–11.19]	1.02 [0.30–3.53]	1.81 [0.76–4.34]	1.08 [0.40–2.65]	0.54 [0.05–5.26]	1.42 [0.99–2.03]
Client Maple score ≥ 4	1.74 [0.95–3.20]	0.84 [0.33–2.18]	1.07 [0.36–3.16]	2.38* [1.42–3.99]	0.91 [0.38–2.21]	0.78 [0.38–1.62]	1.40* [1.06–1.85]
Client CHESS score ≥ 3	1.50 [0.65–3.49]	3.15 [0.95–10.47]	1.05 [0.17–6.39]	4.56** [1.93–10.77]	1.02 [0.47–2.25]	1.10 [0.46–2.67]	1.72* [1.21–2.45]
Bladder incontinence	1.67 [0.82–3.45]	1.16 [0.49–2.79]	1.45 [0.64–3.32]	2.12* [1.29–3.48]	1.23 [0.56–2.70]	1.22 [0.66–2.25]	1.62** [1.24–2.11]
Hospital stay last 90 days	1.90 [0.97–3.71]	2.06 [0.88–4.82]	1.20 [0.42–3.41]	2.32* [1.35–4.01]	1.38 [0.69–2.75]	1.42 [0.61–3.35]	1.71** [1.29–2.27]
Hours of formal care^a^	0.97 [0.94–1.01]	1.10* [1.02–1.18]	0.97 [0.92–1.06]	1.07* [1.01–1.14 ]	1.04 [0.94–1.15]	1.06* [1.01–1.12]	1.02 [0.99–1.04]
Client engaged in ≥ 2 h of physical activities in the last 3 days	1.70 [0.82–3.52]	0.50 [0.11–2.37]	0.42 [0.13–1.35]	0.38* [0.16–0.90]	[–]^b^	1.04 [0.55–1.96]	0.82 [0.58–1.17]

^a^ Hours of formal care, on average, in the last 7 days before the evaluation.

^b^ Only two responses, statistical analysis is impossible.

* *p* < 0.05.

** *p* < 0.001.

If the care recipient was female, the likelihood of caregiver distress decreased in all of the participating countries. In Iceland, Finland and Germany, the likelihood of caregiver distress also decreased when the recipient engaged in at least 2 h of physical activity in the 3 days preceding the evaluation.

## 4 Discussion

This study aimed to determine the prevalence of caregiver distress among home care clients in six European countries and identify which aspects of the client's health, function and conditions predict caregiver distress among informal caregivers. The prevalence of caregiver distress was 34% in Iceland (highest), 28%in Belgium (second highest), 22% in Italy, 17% in the Netherlands, 14% in Germany and 9% in Finland (lowest). In all six countries, enhanced caregiver distress was noted when care recipients experienced depression and deteriorating health, had increased care needs and a live‐in caregiver (Table 3). Results from the different countries studied indicate considerable variations in caregiver distress. The results for Iceland are consistent with findings from New Zealand, where 39.6% of caregivers of home care clients experienced caregiver distress [8]. Similarly, recent studies from Canada have shown a prevalence of caregiver distress of just over 20% for home care [10] and palliative care [27]. The percentages of caregiver distress seen in Finland, Germany and the Netherlands were lower than those published in studies from other countries. In cross‐sectional study of caregivers of older relatives with Alzheimer's or other dementias, the rates of caregiver distress were 15.5% in Hong Kong and 13.9% in New Zealand [30]. It should be noted that, unlike the present study, home care clients who have planned admittance to nursing homes in the next 6 months were included in prior research. This fact may attenuate the reported prevalence of distress in the present study, and therefore, comparisons to previous studies should be made with that caveat in mind.

Comparing the results of this study to the results from the AdHOC study, conducted in the same countries 13 years earlier or in 2001–2002, using the same instrument and the same inclusion and exclusion criteria, the rate of caregiver distress has changed. In the AdHOC study, caregiver distress was higher in Germany (15.1%); slightly lower in Finland (5.3%) and Italy (17.7%); and much lower in the Netherlands (2%) and Iceland (2.6%). It should be noted that the item ‘Family or close friends report feeling overwhelmed by person's illness’ was not included in the AdHOC study [31]. The difference in distress between the two studies in the Netherlands (from 2% to 17%) and especially Iceland is notable, with distress in Iceland increasing to 34%. Such increases indicate that generational attitudes may have changed due to significant social changes. More women are in the labour market and, therefore, face increased demands because they must perform at work, at home and in society [32]. Moreover, the generation that currently has old and even dependent parents may be expected that they want to have more time for themselves and be free to arrange their leisure time independently. The present expectation, especially in Nordic countries, is that formal services will allow older people to live at home longer. Caregivers in these countries assume that the social and health care system will mostly care for their dependent older relatives. Informal caregivers may therefore experience frustration and distress when the services from the formal system do not match that expectation.

This study shows that where the Nordic welfare system is in place in countries such as Iceland and Finland, more hours of formal care were associated with increased caregiver distress. This finding seems contradictory, but the likeliest explanation is that the amount of formal service is insufficient, with only 3.6 h on average weekly in Iceland and 5.1 h in Finland, compared to 7.5 in Germany and 8.5 in Belgium [16]. If an older person has severe needs, this level of formal service may not be enough to ease the burden of care for the informal caregiver, who will, therefore, feel discomfort in the caregiving role. Formal services can decrease caregiver distress, as was seen in Pauley et al.'s study [10], where it appeared that daily visits from a nurse decreased caregiver distress significantly (OR: 0.75).

In previous studies using the same methodology as this study, except that clients who were planning to move to a nursing home in the next 6 months were not excluded, caregiver distress has been associated with physical, mental, cognitive and social conditions [8, 10, 27, 30]. When a primary caregiver lives with an older person, the odds of caregiver distress in Finland, Germany and the Netherlands were significantly greater. In 80%–90% of cases, this caregiver is a spouse. In Canada [10], New Zealand [30] and in the AdHOC study [31], living with the client also increases the likelihood of caregiver distress. In Vaingankar et al.'s study [33], caregiver burden was measured with the Zarit Burden Interview scale, and being married to the care recipient was a predictive factor (OR: 2.4) for experiencing discomfort in caring. Other studies have shown that spouses who experience caregiver distress have poorer health outcomes [30, 34]. These findings indicate that always being on duty and having to respond to all changes and care needs of one's partner are stressful.

In all participating countries, a higher likelihood of caregiver distress was observed when a home care client scored three or higher on the DRS scale. This increased likelihood has also been observed in other studies [8, 35] and the AdHOC study [36]. Caring for an older person with signs of depression can affect a caregiver's mental well‐being. Similarly, physical factors such as bladder incontinence and higher scores on the CHESS scale indicated levels of medical complexity and a risk of a severe decline in health that increased the likelihood of caregiver distress, especially among the Icelandic and Finish caregivers. Scoring 4 or higher on the MAPLe scale, highlighting the need for assistance for home care and predicting long‐term care placement were also a predictive item for caregiver distress in Belgium, Germany and Iceland. This corresponds to results of previously published studies [8, 10, 26, 27].

In Iceland, older persons' hospital visits in the last 90 days increased the likelihood of caregiver distress, but one‐third of home care clients in Iceland who had caregivers with caregiver distress were admitted to the hospital during that time. Those admitted were likely the frailest, and if they returned home before completing recovery, they might have lost their self‐care abilities during the hospital stay. Similarly, if no changes were made to the formal service provided at home after discharge, the family caregiver may have experienced an increased workload. Results from Canada [10] show that hospitalisations in the last 90 days were associated with a lower likelihood of caregiver distress. Hospitalisation was not thought to reduce caregiver distress, but the additional care provided following hospitalisation alleviated it.

Unsurprisingly, the likelihood of caregiver distress reduced in Iceland if an older person had engaged in physical activity for over 2 h in the previous 3 days. This engagement also reduced caregiver distress in Finland and Germany and was consistent with results from Canada [10]. Physical activities require specific physical skills, with mobility reflecting relatively better health. Icelandic health authorities are aware of the importance of physical activity among older people. In a new action plan for services for older people in Iceland (2023–2027) titled *Aging is Good* [37], physical activity is one of the five key elements. The plan highlights actions that promote healthy ageing and require fewer specific services. Facilities for comprehensive mental, physical and social health promotion will be available to increase older people's engagement in physical activities and overall health. With a population of independent older people, families do not need to provide as much care, which could reduce caregiver distress.

Caregivers are important for maintaining older people at home in the community. Therefore, they must feel comfortable in their role and possess the skills and ability to perform it. Caregiver distress can cause informal caregivers to no longer trust themselves in caring for their older family members, meaning that care recipients may not be able to stay at home as long. Awareness of the signs of caregiver distress, for instance, through a comprehensive assessment tool such as interRAI‐HC, and providing caregivers with the necessary support to reduce it is crucial. Research has shown that diverse resources, such as respite care, group support and technology‐based interventions, can reduce caregiver distress [38, 39]. An understanding and knowledge of the factors that cause or prevent caregiver distress are necessary to improve caregivers' health and well‐being. Home care providers are in a superior position during home visits to discuss health and well‐being with informal caregivers and note signs of caregiver distress. However, the caregivers are not usually the focus of home care providers' visits. Therefore, signs of caregiver distress are unlikely to be detected.

Authorities need to develop measures to prevent or reduce the likelihood of distress among caregivers. Providing resources that support caregivers is essential. These resources must be tailored to each caregiver. It is equally important to consider ways of preventing caregivers from becoming overly burdened so that older persons can live at home in a safe environment for as long as possible.

The major strength of this study was its use of the internationally validated and reliable interRAI‐Home Care assessment tool. This tool allows for comparing results across countries and timeframes because the assessment is based on the client's evaluation. The sample from Iceland represents home care clients in the capital area, where over 60% of the country's population lives, but this is not the case with the other countries. A limitation of the study is the lack of information about caregiver characteristics, which could be necessary for understanding the association with caregiver distress. Moreover, the cross‐sectional nature of the data limited its ability to imply causality.

## 5 Conclusion

This study indicates the extent of caregiver distress in several European countries and shows how to identify the factors predicting caregiver distress. Accordingly, the findings help professional caregivers gain greater insight into what informal caregivers are experiencing. Assessment outcomes, such as depression or signs of depression, a decline in health, significant service needs and a recent hospital stay, predict caregiver distress. Using the interRAI‐Home Care assessment tool can be helpful for home care personnel in their observations. Knowledge of these factors can help provide and improve support for caregivers. Home care personnel can identify caregiver distress and provide informal caregivers guidance on relieving their stress. Diagnosing caregiver distress is inconsequential without available resources; establishing resources in the service chain is necessary.

## Author Contributions

All authors conceptualised the study. I.V.K. conducted data collection, analysed the data, and drafted the manuscript. P.V.J. was a co‐investigator in the IBenC study. K.B. supervised the study and co‐wrote the manuscript. All authors revised and approved the final manuscript.

## Conflicts of Interest

The authors, Kristin Bjornsdottir and Ingibjorg Hjaltadottir, declare no potential conflicts of interest with respect to the research, authorship and/or publication of this article. The two other authors, Palmi V. Jonsson and Inga V. Kristinsdottir, are part of the interRAI collaborative network of researchers and practitioners.

## Data Availability

The data supporting this study's findings are not publicly available due to privacy or ethical restrictions.
